# Human papillomavirus infection and risk of breast cancer: a meta-analysis of case-control studies

**DOI:** 10.1186/s13027-016-0058-9

**Published:** 2016-03-14

**Authors:** Jong-Myon Bae, Eun Hee Kim

**Affiliations:** Department of Preventive Medicine, Jeju National University School of Medicine, Jejudo, South Korea

**Keywords:** Breast neoplasms, Risk factor, Human Papillomavirus, Meta-analysis

## Abstract

**Background:**

Although systematic reviews (SR) report that human papillomavirus (HPV) increases the risk of breast cancer, there are still disputes regarding this association. In particular, it has been argued that the risk level differs depending on nationality, type of tissue, subtype of HPV, and publication year. Considering that the searching year of publication for the previous SRs was June 2013, a renewal meta-analysis needs to be conducted.

**Methods:**

Using articles selected in the previous SRs, we compiled a list of references, cited articles, and related articles from the PubMed and Scopus databases. Of these, only publications with data from case-control studies on HPV DNA-positivity in tissues were chosen. Summary odds ratio (SOR) and 95 % confidence interval (CI) were calculated through meta-analysis. Meta-regression analysis was performed for nationality, types of tissue, subtype of HPV, and publication year.

**Results:**

Twenty-two case-control studies were selected, and the total number of individuals in the case and control group was 1897 and 948, respectively. According to the meta-analysis about the 22 publications, HPV infection increased the risk of breast cancer (SOR = 4.02, 95 % CI: 2.42–6.68; I-squared = 44.7 %). Statistical significance was not found in meta-regression performed on the four variables of nationality, type of tissue, subtype of HPV, and publication year which some researchers think sources of heterogeneity.

**Conclusions:**

The results of the present study supported the argument that HPV infection increases the risk of breast cancer. Age-matched case-control studies are in need in the future.

## Background

Breast cancer is a primary cancer that has one of the highest incidences in women worldwide [1,2]. Epidemiologically, breast cancer occurs at a younger age for Asians compared to individuals from western countries [3]. Similarly, the peak incidence of breast cancer among Korean women occurs around 45–49 years old, which is immediately before menopause, and decreases after this age, forming a single-peak curve [4]. Because the shape of this curve had been maintained for the last 20 years, it was reported that there was no cohort effect in age-period-cohort analysis [5]. Based on the epidemiological characteristics mentioned above, dense breast and human papillomavirus (HPV) were proposed as risk factors related to the development of breast cancer among Korean women [6,7].

In particular, the HPV infection theory is significant in the prevention of cancer because HPV vaccines are currently in use [8]. The association between HPV infection and breast cancer was first proposed in 1992 [9], and since then there was a report of HPV DNA being detected in breast cancer tissues of Korean women [10]. And three meta-analyses [11–13] reported that HPV DNA was detected in 23–30 % of breast cancer tissues and the summary odds ratio (SOR) was 3.24–3.63 with statistical significance.

Nevertheless, there are still disputes about the association between HPV infection and the risk of breast cancer [14]. In particular, there is debate over the use of paraffin-embedded tissue (PET) to test for HPV DNA-positivity because HPV DNA can be destroyed and become contaminated during the treatment procedure, meaning that PET will have more measurement errors than fresh frozen tissue (FFT) [15]. Although Li et al. [12] emphasized that HPV 33 was detected in all Asians, it was suggested that these regional differences can be attributed to differences in the testing method [15]. In addition, Zhou et al. [13] stressed that the risk of HPV infection was influenced by geographic region, HPV DNA source, PCR primer used, and publication year. However in the subgroup analysis, the confidence intervals of SOR overlapped with one another. Therefore, it is necessary to further examine whether these variables indeed cause heterogeneity. Furthermore, taking into account that the final search period of the 3 SRs was June 2013 [11], the meta-analysis needs to be adapted by additionally selecting literatures published up to September 2015. The objective of this study was to re-conduct meta-analysis with meta-regression on the relationship between HPV infection and the risk of breast cancer.

## Results and discussion

**Fig. 1:**
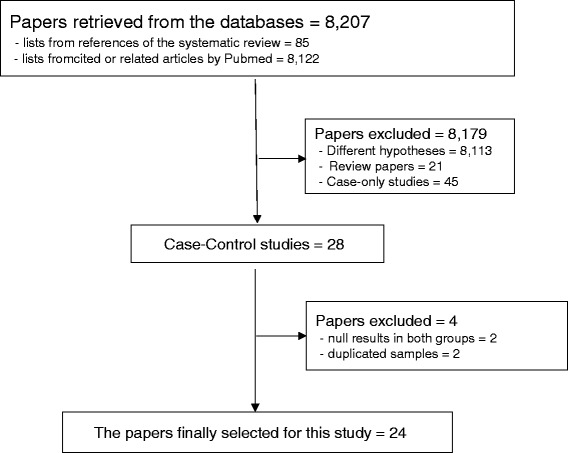
Flow chart of article selection

Figure 1 depicts the process of selecting articles for the final analysis through a data search. Based on the 3 SRs to identify the association between HPV and the prevalence and odds ratio (OR) of breast cancer, a list was compiled containing 85 references and 8122 cited and related articles from PubMed and Scopus. We sequentially applied the selection criteria into the total 8207 papers, and excluded (1) 8113 articles with a different hypothesis, (2) 21 articles that were expert reviews or systematic reviews, (3) 45 articles using case only studies, (4) 2 articles that were case-control studies without HPV DNA-positivity in both groups [16,17], and (5) 2 articles published using duplicate samples [18,19]. The older publication in 2005 by Tsai et al. [18] was excluded because the samples used were the same as a publication in 2007 [20] by the same group. In addition, the studies published in 2009 by Lawson et al. [19] and Hang et al. [21] used the same DNA specimens as each other; of these, Lawson et al. [19] was excluded based on the suitability of the hypothesis for our study.

Following the aforementioned exclusion process, 24 publications were selected for the meta-analysis [10,14, 20–41]. Table 1 summarizes the numbers of HPV DNA-positive and HPV DNA-negative individuals in the case and control group in these 24 case-control studies, organized according to the nationality of the study subjects, types of DNA specimen, and 3 HPV subtypes. Of these studies, He et al. [28] and Fu et al. [40] used the same DNA specimens. Therefore, Fu et al. study published in 2015 [40] was used for the overall analysis, and He et al. study published in 2009 [28] was used only for analyzing the HPV 16 results. For similar reasons, data from Glenn et al. published in 2012 [33] was used for the overall and HPV 18 analyses, while the data from Heng et al. published in 2009 [21] was used for analyzing the HPV 16 results. Therefore, in the 22 publications of case-control studies excluding the 2 articles that used DNA specimens from the same hospital [21,28], there were 1897 and 948 individuals in the case and control group, respectively. When categorized by region, there were 10 articles in far-east Asia, 5 articles in middle-east Asia, and 7 articles in other regions. By specimen type, there were 15 articles using PET and 7 articles using FFT. When the data was organized by HPV subtype, there were 11 articles on HPV 16, 10 articles on HPV 18, and 5 articles on HPV 33.

**Table 1:** Summary of the selected case-control studies by subtypes of human papillomavirus*

First author	Year of publication	Reference number	Nation	Tissue type*	Any type		16 Subtype		18 Subtype		33 Subtype	
					Case (n/N)	Control (n/N)	Case (n/N)	Control (n/N)	Case (n/N)	Control (n/N)	Case (n/N)	Control (n/N)
Yu	1999	22	China/Japan	PET	18/52	1/20					18/52	1/20
Damin	2004	23	Brazil	PET	25/101	0/41	14/101	0/41	10/101	0/41		
de Villiers	2005	24	USA	PET	25/29	20/29						
Gumus	2006	25	Turkey	FFT	37/50	16/50			20.37	9/16	35/37	14/16
Choi	2007	10	Korea	PET	8/123	0/31						
Tsai	2007	20	China	FFT	8/62	2/32						
Khan	2008	26	Japan	PET	26/124	0/11	24/124	0/11	3/124	0/11	1/124	0/11
de Leon	2009	27	Mexico	PET	15/51	0/43	10/51	0/43	3/51	0/43		
Mendiazabauiz	2009	29	Mexico	PET	3/67	0/40						
Herrera-Goepfert	2011	41	Mexico	PET	6/60	7/60	6/60	7/60				
Mou	2011	30	China	FFT	4/62	0/46	3/62	0/46	1/62	0/46		
Chang	2012	31	China	FFT	0/48	3/30						
Frega	2012	32	Italy	PET	9/31	0/12						
Glenn	2012	33	Australia	FFT	25/50	8/40			25/50	8/40		
Sigaroodi	2012	34	Iran	PET	15/58	1/41	4/79	0/51	4/79	0/51		
Liang	2013	35	China	FFT	48/224	6/37						
Ali	2014	36	Iraq	PET	60/129	3/44	33/129	0/44	35/129	1/44	16/129	1/44
Ahangar-Oskouee	2014	37	Iran	PET	22/65	0/65	1/65	0/65				
Manzouri	2014	38	Iran	PET	10/55	7/51	2/55	0/51	1/55	0/51	1/55	0/51
Peng	2014	39	China	FFT	2/100	0/50			2/100	0/50		
Fu	2015	40	China	PET	25/169	1/83						
Li	2015	14	China	PET	3/187	0/92						
He	2009	28	China	FFT			24/40	1/20				
Heng	2009	21	Australia	PET			1/26	0/17				

*(*n/N*) number of positive on the tested samples, *FFT* fresh frozen tissue, *PET* paraffin-embedded tissue

**Fig. 2:**
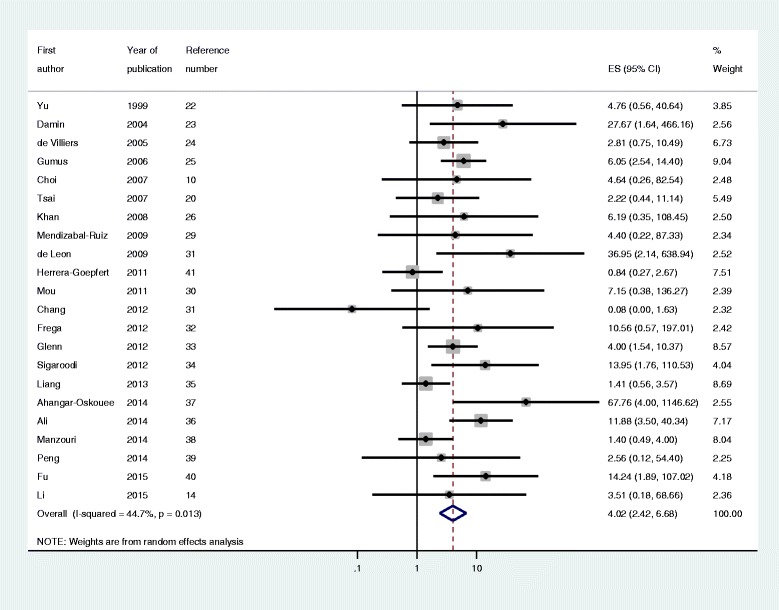
The forest plot of using a random‐effects summary estimates in 22 case‐control studies. ES : effect size; CI: confidence intervals

**Fig. 3:**
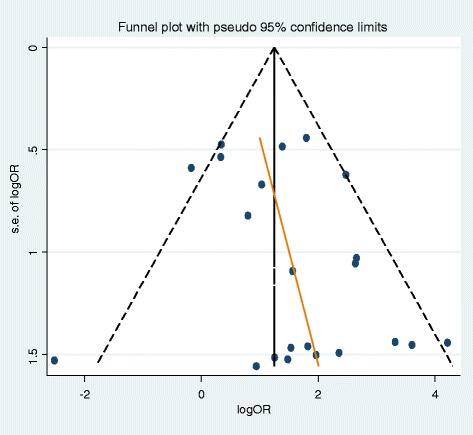
The funnel plot of using a mixed‐effects summary estimates in 22 articles (*P*‐value of Egger test =0.165). LogOR: log odds ratio; s.e.of logOR: standard error of log odds ratio

Regardless of HPV subtype, the risk of breast cancer was 4.02-fold higher (95 % CI: 2.42–6.68: I-squared =44.7 %) for HPV DNA-positive individuals (Fig. 2). The Egger test was used to determine publication bias, and the bias coefficient was 0.91 which was not statistically significant (*p* = 0.165) (Fig. 3).

Table 2 summarizes the results of subgroup analysis by HPV subtype, region, and type of DNA specimen. Results by region showed that risk of breast cancer for HPV DNA-positive individuals was 7.04-fold higher in middle-east Asia (95 % CI: 2.43-20.42), 4.23-fold higher in America regions (95 % CI: 1.06-16.84), and 2.60-fold higher in far-east Asia (95 % CI: 1.25-5.38). By specimen type, the risk was 5.60-fold higher for PET (95 % CI: 2.79-11.25) and 2.61-fold higher for FFT (95 % CI: 1.22-5.61). Although there were differences in SOR by region, specimen type and publication periods, all risks were statistically significant. However, the CIs of SORs all overlapped.

**Table 2:** Subgroup analyses by subtypes of human papillomavirus

Types	Sub-group		Numbers	I-squared (%)	SOR with 95 % CI by REM
Any			22	44.7	4.02 [2.42–6.68]
	Nationality of subjects	Far-East	10	16.4	2.60 [1.25–5.38]
		Middle-East	5	66.4	7.04 [2.43–20.42]
		America	5	59.2	4.23 [1.06–16.84]
		Europe & Oceania	2	0.0	4.39 [1.77–10.86]
	Specimen types	PET	15	45.4	5.60 [2.79–11.25]
		FFT	7	47.5	2.61 [1.22–5.61]
	Year of publication	−2010	9	0.0	5.10 [2.89–8.99]
		2011-	13	60.1	3.55 [1.68–7.50]
16			11	32.5	5.67 [2.21–14.52]
	Nationality of subjects	Far-East	3	0.0	12.37 [2.83–54.15]
		Middle-East	4	0.0	7.87 [1.76–35.23]
		America	4	54.3	3.58 [0.61–21.03]
	Specimen types	PET	9	25.9	4.25 [1.57,11.54]
		FFT	2	0.0	16.47 [2.94–92.18]
	Year of publication	−2010	5	0.0	12.47 [3.73–41.71]
		2011-	6	30.1	3.25 [0.97–10.87]
18			10	0.0	2.97 [1.64–5.38]
	Nationality of subjects	Far-East	3	0.0	1.54 [0.26–9.20]
		Middle-East	4	52.5	3.37 [0.70–16.22]
		America	3	0.0	4.50 [1.89–10.68]
	Specimen types	PET	7	28.0	3.13 [1.09–9.03]
		FFT	3	0.0	3.70 [1.54–8.88]
	Year of publication	−2010	4	12.4	1.59 [0.51–4.96]
		2011-	6	0.0	4.62 [2.17–9.81]
33			5	0.0	3.64 [1.26–10.48]
	Nationality of subjects	Far-East	2	69.6	2.10 [0.06–68.51]
		Middle-East	3	0.0	3.70 [0.98–13.90]
	Specimen types	PET	5	0.0	3.64 [1.26–10.48]
		FFT	-	-	-
	Year of publication	−2010	3	40.4	2.64 [0.44–15.76]
		2011-	2	0.0	4.88 [0.87–27.56]

*CI*confidence intervals, *FFT* fresh frozen tissue, *PET* paraffin-embedded tissue, *REM* random effect model

When we examined the results by HPV subtype, the risk of breast cancer was, in descending order, 5.67-fold higher for HPV 16 (95 % CI: 2.21-14.52), 3.64-fold higher for HPV 33 (95 % CI: 1.26-10.48), and 2.97-fold higher for HPV 18 (95 % CI: 1.64-5.38), and all risks were statistically significant. Again, the CIs of SORs were overlapping for 3 subtypes.

The meta-regression analysis was performed on 26 datasets created around three subtypes, with nationality, types of tissue, subtype, and publication year as the variables. None of the variables showed statistical significance (not shown).

In order to satisfy the criteria to prove that a specific virus causes cancer [42], case-control studies must be performed instead of case only studies [43]. However, tumor-based case-control studies are susceptible to measurement errors [44,45], and thus, systematic reviews are needed to overcome this shortcoming.

According to the meta-analysis for results from 22 case-control studies, the risk of breast cancer due to HPV infection was 4.02-fold higher. Even when the results were analyzed by categorizing into four regions, two types of DNA specimen and two publication periods, the risk of breast cancer due to HPV was statistically significant. The findings provide supporting evidence for the HPV infection as a risk factor of breast cancer. Additionally, the CIs of SOR calculated in the subgroup analysis were overlapping with one another, and the results from meta-regression analysis showed that none of the 4 variables caused heterogeneity. These findings support the validity of the SOR calculated in the meta-analysis.

The estimated SOR in this study was similar to previous meta-analysis results (Table 3). However, our meta-analysis retrieved results from 22 case-control studies, and therefore, has a narrower confidence interval because we were able to retrieve publications that were not selected through electronic search. The list of 22 publications gathered in this manner will be important for renewal meta-analyses in the future.

**Table 3:** Comparison of three meta-analyses for HPV infection and breast cancer risk

First author [reference number]	Searching Periods	Selected articles	Total Cases	Total Controls	SOR (95 % CI)
Li [12]	- May 2010	10	447	275	3.63 (1.42–9.27)
Simoes [11]	- Jan 2011	9	448	279	5.9 (3.23–10.67)
Zhou [13]	- Jun 2013	16	n.d.	n.d.	3.24 (1.59–6.57)
This study	- Sep 2015	22	1897	948	4.02 (2.42–6.68)

*n.d* not described

Early study results were confusing, due to inappropriate experimental design, small sample sizes, and unstandardized HPV DNA detection methods [11,14,15]. However, Li et al. [12] commented that consistent study results have been reported since 2006. Therefore, we tried to conduct a subgroup analysis by dividing into before and after 2006, but because only 3 of the 21 publications were before 2006, we performed analysis with 2010 as the cut point. In terms of selecting region variables, 9 out of 16 studies selected in Zhou et al. [13] had Asian subjects, whereas in this study it was 15 out of 22 studies that had Asian subjects. Thus, in the study, an analysis was done after the 15 studies were separated into 10 far-east and 5 middle-east Asia studies. Also, Zhou et al. [13] reported the difference for each PCR primer even if the CIs of SORs overlapped. In this study, we used the subtype variable, in lieu of the variable of PCR primer used. That is, we created 26 sets of database after dividing HPV into 3 subtypes (16, 18, and 33) and examined SOR by subtype. Not only the results showed that the CIs of SOR calculated by subtype overlapped, but also we confirmed no statistical significance with a meta-regression analysis.

Regarding the link between the Epstein-Barr virus infection and breast cancer, it has been argued that different kinds of control tissue cause heterogeneity [46]. Of the 22 selected studies, we found that only 2 studies used adjacent normal cells from the cancer tissue [24,41], and the remaining 20 studies used normal breast cells of non-cancer tissues. Therefore, an additional analysis by type of control tissue was not performed.

It has been proposed that not only HPV but also herpesvirus, polyomavirus, and beta retrovirus increase the risk of breast cancer [47]. Proving these theories related to viral infection is of great significance because it opens up the possibility of using antiviral drugs to treat breast cancer and vaccines to prevent breast cancer [8,48].

## Conclusions

In conclusion, this meta-analysis supports the hypothesis that HPV infection is a risk factor for breast cancer. In near future, it is anticipated that nested case-control studies will be actively performed, along with age-matched case-control studies.

## Methods

### Search and selection of related articles

Since we were using 3 previously published systematic reviews [11–13], we used the hand search method rather than the electronic search method [49,50]. Publications were found by searching the references of articles selected in these 3 systematic reviews on the preferential basis. And then lists of “cited articles” and “similar (related) articles” provided by the PubMed (www.ncbi.nlm.nih.gov/pubmed) and Scopus (www.elsevier.com/solutions/scopus) databases for each article were also considered for inclusion. This searching strategy assumes that studies conducted with the ‘same research hypothesis’ have a high possibility of being cited in related articles and that they will have similar findings [51].

The final selection criteria were case-control studies that detected HPV DNA in the tissue. Based on the titles and abstracts for the papers in the compiled list, the following 5 exclusion criteria were applied sequentially. (1) Articles with different hypothesis, (2) expert reviews or systematic reviews, (3) case only studies, (4) case-control studies without HPV DNA-positivity in both groups, and (5) articles published by using the same DNA samples as another study. The remaining case-control studies after applying the 5 aforementioned criteria were selected as publications for the final analysis.

### Statistical analysis

Two researchers applied the exclusion criteria for each publication and retrieved HPV-related data—the number of HPV DNA-positive and HPV DNA-negative individuals in the case and control group, nationality of study subjects, types of DNA specimen, types of HPV subtypes, and publication period. Using the obtained number of HPV DNA-positive and HPV DNA-negative individuals in the case and control group, OR and 95 % CI were calculated for each article. Based on the prevalence of HPV subtypes reported by the Zhou et al. [13], data on high-risk type-specific HPV 16, 18, and 33 were organized separately. Based on the nationality of study subjects, groups were categorized into far-east Asia (Korea, China, and Japan), middle-east Asia (Turkey, Iran, and Iraq), America, and Europe & Oceania regions. Specimen types were classified into PET and FFT groups. Publication year was divided into 2 groups with 2010 as the cut point.

The presence of heterogeneity in meta-analysis was assessed using the I-squared value (%). The summary odds ratio (SOR) for a random effect model and its 95 % CI were calculated first because if the I-squared value is 0.0 %, using either a random effect model or a fixed effect model will result in the same value. To determine the publication bias, Egger’s test for small-study effects was conducted [52]. Additionally, a subgroup analysis and a meta-regression analysis were conducted using the 4 potential variables thought to cause heterogeneity in risks—geographic region, HPV DNA source, publication year, and subtype of HPV. *P*-value of less than 5 % was considered statistically significant, and STATA version 14 (www.stata.com) statistics program was used.
